# "Electro-clinical syndromes" with onset in paediatric age: the highlights of the clinical-EEG, genetic and therapeutic advances

**DOI:** 10.1186/1824-7288-37-58

**Published:** 2011-12-19

**Authors:** Pasquale Parisi, Alberto Verrotti, Maria Chiara Paolino, Rosa Castaldo, Filomena Ianniello, Alessandro Ferretti, Francesco Chiarelli, Maria Pia Villa

**Affiliations:** 1 NESMOS Department, Chair of Pediatrics, Child Neurology, Faculty of Medicine and Psychology,, "Sapienza" University, Via di Grottarossa, 1035-1039, Rome,00189, Italy; 2 Department of Paediatrics, University of Chieti, Ospedale policlinico SS. Annunziata, Via dei Vestini 5, 66100 Chieti, Italy

**Keywords:** Electro-clinical syndrome, Paediatric Epilepsy, EEG, Epileptic Syndrome, ILAE classification

## Abstract

The genetic causes underlying epilepsy remain largely unknown, and the impact of available genetic data on the nosology of epilepsy is still limited. Thus, at present, classification of epileptic disorders should be mainly based on electroclinical features. Electro-clinical syndrome is a term used to identify a group of clinical entities showing a cluster of electro-clinical characteristics, with signs and symptoms that together define a distinctive, recognizable, clinical disorder. These often become the focus of treatment trials as well as of genetic, neuropsychological, and neuroimaging investigations. They are distinctive disorders identifiable on the basis of a typical age onset, specific EEG characteristics, seizure types, and often other features which, when taken together, permit a specific diagnosis which, in turn, often has implications for treatment, management, and prognosis. Each electro-clinical syndrome can be classified according to age at onset, cognitive and developmental antecedents and consequences, motor and sensory examinations, EEG features, provoking or triggering factors, and patterns of seizure occurrence with respect to sleep. Therefore, according to the age at onset, here we review the more frequently observed paediatric electro-clinical syndrome from their clinical-EEG, genetic and therapeutic point of views.

## Introduction

Syndrome classification is not applicable to all patients with epilepsy, but only a limited number of patients [1]. In fact, the 1989 syndrome classification assigned, in each category, "other epilepsies not defined as syndromes", using, moreover, the terms ''syndromes'' and ''epilepsies'' almost interchangeably. The result was that the term ''syndrome'' took on a broad and very imprecise meaning, to the point where very specific and highly recognizable entities (such as childhood absence epilepsy) and poorly differentiated and not well-described epilepsies (such as cryptogenic parietal lobe epilepsy), tended to be treated as though they represented the same level of diagnostic precision.

Moreover, in the past, the term ''idiopathic'' was also used to convey the idea of a highly pharmaco-responsive form of epilepsy, whereas, in the new ILAE terminology, the implication that ''idiopathic'' confers the quality of ''benign'' is, now-days, intentionally discarded. Thus, as a variety of subtle cognitive and behavioral disorders are seen in association with idiopathic epilepsies, cause is no longer equated with prognosis.

In this respect, in fact, at the present time, it might be reasonable to include some of the traditional electro-clinical syndromes (ECS) (such as, benign rolandic epilepsy, Panayiotopoulos syndrome, and benign occipital epilepsy of the Gastaut type), previously classified as ''idiopathic'', in the unknown category [2].

In addition, correlations between genotype and phenotype in ECS (table 1) are not so easy to establish, since genetic and non genetic factors likely play a role in determining the severity of ECS phenotype. On the other hand, the numerous and increasing discoveries on genetic origin are improving our knowledge, and it is hoped that this will be useful to define a more targeted therapeutic approaches for at least some of ECS.

**Table 1:** Electro-clinical syndromes in paediatric age according to age at onset

**A) Neonatal period:** < 44 weeks of gestational age	BFNE EME Ohtahara syndrome
**B) Infancy:** < 1 year	Epilepsy of infancy with migrating focal seizures West syndrome MEI Benign infantile epilepsy Benign familial infantile epilepsy Dravet syndrome Myoclonic encephalopathy in non progressive disorders FS+ (can start in childhood)
**C) Childhood:** 1-12 years	FS+ (can start in infancy) PS Epilepsy with myoclonic atonic (previously astatic) seizures BCECTS ADNFLE Late onset childhood occipital epilepsy (Gastaut type) Epilepsy with myoclonic absences Lennox-Gastaut syndrome CSWS LKS CAE
**D) Adolescence:** 12-18 years	JAE JME Epilepsy with generalized tonic-clonic seizures alone Familial focal epilepsy with variable foci (childhood to adult) Reflex epilepsies

BFNE: Benign familial neonatal epilepsy; EME: Early myoclonic encephalopathy; MEI: Myoclonic epilepsy in infancy; FS+: Febrile seizures plus; PS: Panayiotopoulos syndrome; BCECTS: Benign epilepsy with centrotemporal spikes; ADNFLE: Autosomal-dominant nocturnal frontal lobe epilepsy; CSWS: Epileptic encephalopathy with continuous spike-and-wave

during sleep; LKS: Landau-Kleffner syndrome; CAE: Childhood absence epilepsy; JAE: Juvenile absence epilepsy; JME: Juvenile myoclonic epilepsy.

One of the most distinctive and clinically salient dimensions for classifying ECS is based accordingly to their typical age at onset, as reported in table 1.

To provide epileptologists a comprehensive highlights of ECS aspects, among the currently ILAE recognized ECS (table 1), here we review, from an "electro-clinical", therapeutics and genetic points of view, the most frequently observed forms with onset in paediatric age (table 1).

### Early Infantile Epileptic Encephalopathy (EIEE) or Ohtahara Syndrome (OS)

**Figure 1:**
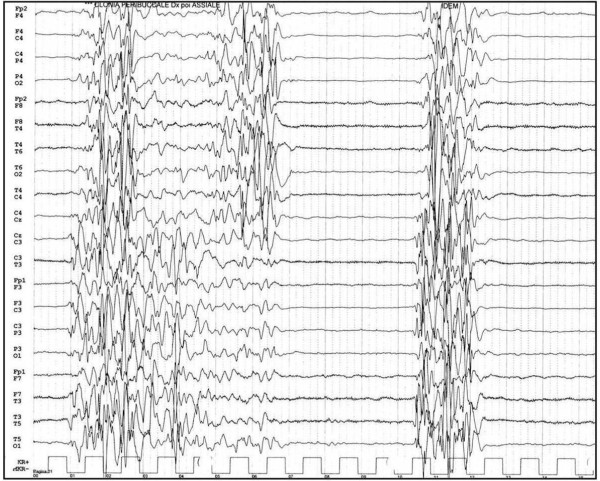
**EEG pattern of suppression burst**.

Early-infantile epileptic encephalopathy, also known as Ohtahara syndrome [3], is characterized by early onset of tonic spasms occurring with or without clustering, seizure intractability, a characteristic interictal bursts-suppression (SB) pattern (Figure 1) on EEG persistently observed in both waking and sleeping states and a remarkable age-dependent evolution into the West syndrome (reported in 75% of cases) [4]. Prognosis is very poor with severe drug-resistance and psychomotor retardation.

The causes of EIEE are heterogeneous. Several brain malformations, neuronal migration disorders and metabolic disorders have been found as underlying causes of symptomatic OS [5]. Two causative genes are thought to be involved in the pathogenesis of cryptogenic cases of OS: the aristaless-related homeobox (ARX) and the syntaxin binding protein 1 (STXBP1) genes. The ARX gene is considered to have an important role in the neuronal proliferation, in differentiation of the embryonic brain, and in interneuronal migration, acting as a transcription factor in the development of GABAergic interneurons [6]. Phenotypes associated with ARX mutations include both malformative and non malformative syndromes.

Pleiotropic mutations of the ARX gene cause a variety of phenotypes that are considered to share a common pathological mechanism related to the structural and functional disturbance of interneurons, called "interneuronopathies" [7,8]. This hypothesis was supported by experimental data demonstrating that ARX protein deficiency results in the loss of GABAergic interneurons and anomalous distribution of residual cells in the cortex and basal ganglia [9]. Consequently, the GABAergic network dysfunction seems to play a crucial role in the pathogenesis of SB and in the hypsarrhythmic pattern. STXBP1, or MUNC18-1 gene encodes syntaxin binding protein 1, a neuron-specific protein that is essential for synaptic vesicle release [10,11]. A recent study showed that mutations in STXBP1 are not limited to patients with OS, but are also present in patients with an early-onset epileptic encephalopathy which do not fit into either OS or WS. This strongly supports the hypothesis that mutations in STXBP1 could cause a many kinds of epileptic disorders [12,13].

Although EIEE is usually characterized by drug resistance, some specific treatments have been reported to be effective, such as ketogenetic diet [14], liposteroid administration [15], zonisamide (ZNS) [16], high-dose phenobarbital (PB) [17], levetiracetam (LEV) [18], and vigabatrin (GVG) [19]. Selected patients could benefit from epilepsy surgery [20].

A similar syndrome, Early Myoclonic Encephalopahty (EME) has an onset within the first few weeks of life. EME has been associated primarily to metabolic disorders while EIEE is more likely associated to structural brain abnormalities. This syndrome differs from EIEE for the main type of clinical seizure observed: EME patients have fragmentary myoclonus while EIEE patients have epileptic spasms. EEG in EME shows a suppression-burst pattern less persistent than the one seen in EIEE. [3].

### Benign Familial Neonatal (BFNS) or Infantile (BFIS) or Neonatal-Infantile (BFNIS) Seizures

BFNS (also known as Benign Familial Neonatal Convulsions) is a rare, monogenic, autosomal-dominant, benign familial epilepsy syndrome [21]. It is characterized by unprovoked and brief cluster of focal tonic-clonic convulsions occurring within the first days of life and frequently flowing into status epilepticus. No specific EEG trait characterizes BFNS: interictal EEG is most commonly normal, and if present, anomalies are usually transient [1,22]. The majority of individuals with BFNS can be kept seizures-free by using PB. Seizures disappear spontaneously within 2 months of life. Hovewer, about 10%-15% of children with BFNS develop seizures later in life with a variable age of onset and duration; in this eventuality, seizures are mainly generalized tonic or tonic-clonic seizures, and EEG may be characterized by centro-temporal spikes and sharp waves or benign epilepsy with centrotemporal spikes [23].

BFNS is linked to mutations in KCNQ2 and KCNQ3 genes [24], which are members of a family of voltage-gated potassium channel genes (KCNQ1-5). They encode the voltage-gated Kv7.2 and Kv7.3 channels that produce a neuronal M-current (muscarinic-regulated potassium current) which limits the repetitive firing of many neurons. More than 60 mutations have been described in BFNS families, with the majority involving KCNQ2.

No major phenotypic differences are observed between patients with BFNS caused by a KCNQ2 mutation and those with BFNS caused by a KCNQ3 mutation. Penetrance is incomplete (85%); anticipation has not been observed [21]. The majority of newborns diagnosed with BFNS have an affected parent; however, also sporadic BFNS have been reported. In BFNIS, seizures appear in neonates, and in BFIS they begin between the 3rd and the 12th month of life.

Most of children with BFNS can keep seizure free by using PB (20 mg/kg as loading dose and 5 mg/kg/day as maintenance dose). In some cases have been used other anti-epileptic drugs (AEDs) like carbamazepine (CBZ), phenytoin (PHT), valproic acid (VPA), clonazepam (CZP), midazolam and GVG. Retigabine, selectively enhancer of potassium channels, is being tested as an adjunctive therapy in individuals with partial onset seizures [25]. Treatment is theoretically unnecessary for BFNIS and BFIS, but as seizures appear in cluster, it may be necessary to treat them. BFNIS condition could have some clinical and genetic features overlapping with BFIS, which is a dominant idiopathic epilepsy with partial and secondarily generalized seizures with age of onset between 3 and 12 months. Moreover, a mechanism that can explain that epileptic spells in BFNIS occur almost exclusively during the first days to months of life has recently been proposed [26]. BFIS is a genetically heterogenous condition with loci mapped to chromosomes 19 and 16. Mutations in the voltage-gated sodium channel alpha2 subunit (SCN2A) gene on chromosome 2 were recently identified in families affected by neonatal and infantile seizures (benign familial neonatal-infantile seizures, BFNIS) with typical onset before 4 months of life. Striano et al reported a novel SCN2A mutation in family with benign familial infantile seizures describing three affected individuals over three generations. Genetic study in this family revealed a novel heterozygous mutation c.3003 T > A in the SCN2A gene. Comparative analysis of different sodium channel alpha subunits indicates that the mutated residue is highly conserved throughout the evolution, suggesting an important functional role for this domain. The identification of SCN2A mutations in families with only infantile seizures indicated that BFNIS and BFIS show overlapping clinical features [27].

### West Syndrome and Infantile Spasms

West syndrome (WS), also called Infantile Spasms (IS) or Salaam Spams/Tics, show an incidence which is estimated about 0,16-0,42 per 1000 live births [28]. In the first description, it was characterized by a famous triad that consists of seizures (so-called IS: generally consisting of, sudden, bilaterally and symmetrical, flexor, extensor or mixed type, spasms of the neck, trunk and extremities), characteristic abnormalities to the electroencephalography (hypsarrhytmia) and psychomotor retardation. The causes of this syndrome are heterogeneous and it can be divided in three principal groups: symptomatic, cryptogenetic and idiopathic. In the symptomatic group the epileptic desease is associated with various brain damage due to prenatal, perinatal and postnatal causes.

WS occurs in the first year of life, with a peak age at 5 months, seizures are heterogeneous and occur at awakening and during crying [29].

In 1950 Gastaut and co-workers described the EEG features associated with WS and then Gibbs and others conied the term hypsarrhythmia to define an EEG characterized by "random high voltage slow waves and spikes, that vary from moment to moment in duration and in location: at the onset, they appear to be focal and then they seem to originate from multiple foci; in few cases the spike discharge becomes generalized" [30].

Stromme and Weaving found mutations of two genes, ARX and STK9, in patients with X-linked familial West syndrome [31,32]. A polyalanine expansion mutation of the ARX gene has also been found in a patient with sporadic cryptogenetic WS [33]. A new STXBP1 mutation on cromosome 9q34.11 in a patients affected by WS without transition from OS has been reported [34]. Recently, a de novo deletion of 16p13.11 (previously suggested as risk factor for mental retardation and multiple congenital anomalies), in a patient with significant developmental delay, facial dysmorphism and WS, has been reported [35].

Many new therapeutic options have been tried: GVG, ZNS, nitrazepam (NZP), methisergide, LEV, topiramate (TPM), lamotrigine (LTG), pyridoxine, ketogenic diet, immunoglobulin therapy, felbamate (FLB), and thyrotropin-releasing hormone [36,37]. ACTH is generally estimated to be more effective than corticosteroids and it appears to positively alter long-term prognosis in the criptogenetic cases more than in the symptomatic ones [38].

GVG has been considered the drugs of first choice for patients affected by IS associated with tuberous sclerosis, as well as with Focal Cortical Dysplasia (FCD), and may also be a second-line agent for the treatment of other symptomatic IS. The efficacy of GVG is increased with high doses (100-150 mg/Kg/day) compared with the standard doses (40-100 mg/Kg/day) [39]. Unfortunately, GVG- related rethinopathy represents a limit for its use [40,41]. Brief period and low dose to minimize the probability of visual field defect GVG related has been suggested [42] and, in case of lack of improvement, GVG should be discontinued after 12 weeks [43].

### Dravet Syndrome (DS) and Genetic Epilepsy with Febrile Seizure Plus (GEFS+)

GEFS+ is a familial autosomal-dominant epileptic syndrome with a large pattern of intra and extrafamilial phenotypic variability. Patients with GEFS+ may suffer from febrile seizures (FS) above the 6th year of age (called febrile seizures plus, FS+) and also afebrile myoclonic, absence, atonic, or partial seizures may appear [44]. FS and FS+ represent the milder form of GEFS+, whereas "severe myoclonic epilepsy of infancy" (SMEI) represent the most severe form. SMEI is an epileptic encephalopathy starting during the first year of life, especially around the 6th month, with recurrent and long-lasting febrile seizures, also as febrile status epilepticus. Drug-resistant myoclonic, complex partial, and atypical absence seizures can appear after the 12 months of life. Hot water seizures and photosensitivity are present in about 50% of the patients [44]. Regression of the normally acquired mental capacities may start from the 2nd year, often related to the episodes of status epilepticus [44]. Interictal myoclonus, ataxia and piramidal signs may complete the clinical picture. Japanese authors described patients with uncomplete SMEI phenotype, and have named these variants as borderline severe myoclonic epilepsy of infancy (SMEB) and intractable childhood epilepsy with generalized tonic-clonic seizures (ICE-GTC) [45,46]. In GEFS+, SMEI, SMEB, and IGE-GTC mutations in the voltage gated sodium channel alpha1 subunit (SCN1A) gene have been discovered. SCN1A gene (Chromosome 2q24.3) is mainly expressed in the cerebral tissue and is implicated in generation and propagation of action potentials [47]. About 10% of GEFS+ patients have SCN1A mutation. In SMEI patients mutations falling in the encoding exons, which are de novo in 95%, are present in about 80% of cases, and they include missense (39%), nonsense (22%), frameshift (19%), splice site (10%), genomic rearrangements (deletions, duplications, amplifications, translocations) (7%), in-frame deletions (2%), and other types (silent, complex mutations; 1%) of mutations [48,49].

Till now, in Dravet syndrome, hundreds of mutations were found in SCN1A, while only few mutations were identified in the paralogue gene, SCN2A, which encodes the alpha2 subunit, that is associated with BFNIS and BFIS, and other various intractable childhood epilepsies [50,51]. Mutations of SCN1B gene (voltage-gated sodium channel beta1 subunit) and GABRG2 gene (GABA receptor gamma2 subunit) were identified in a few families with GEFS+ spectrum [52,53].

A number of AEDs such as VPA, clobazam (CLB), TPM and LEV, are reported to be efficacious in DS [54,55]; VPA is used as a first-line agent to prevent the recurrence of febrile seizures and oral/nasal/rectal benzodiazepine is used for any long-lasting seizures, but these agents are most often insufficient. LTG, CBZ, and high doses of intravenous PB should be avoided because they may worsen seizures. TPM, LEV, bromide, and the ketogenic diet may provide substantial efficacy as adjunctive therapy/procedure [56]. Chiron reported the efficacy of stiripentol, a P450 inhibitor, used with VPA and a benzodiazepine [57]. Alternative therapies such the ketogenic diet, with controversial results, have been tried [54,55].

Prolonged treatment with sodium channel-inhibiting AEDs may aggravate the disease course or lead to severe refractory status epilepticus [58,59].

### Early-Onset Absence Epilepsy (EOAE)

Absence seizures are epileptic manifestations that may start in children typically between the 4^th ^and the 10th year of age (Childhood Absence Epilepsy, CAE). Less commonly, absence seizures may arise before the 4th year of age (Early-onset Absence Epilepsy), and may be associated with other neurological disorders (other types of seizures, developmental delay, and movement disorders) [60]. Recently it has been suggested that Early-onset pure absence epilepsy (EOAE) is a distinct epilepsy characterized by absences starting from a few months to 4 years of age, normal early psychomotor development, good AEDs seizure control and normal intellectual outcome [61].

Mutations in three different genes have been reported in children with absence seizures: anomalies of GABRG2 gene have been described in patients with febrile seizures and CAE [62,63], and mutations in SCN1B and SCL2A1 genes have been reported in children with EAOE with [64] or without [65] febrile seizures. SCL2A1 gene encodes glucose transporter type 1 (GLUT1), a glucose transporter across the blood-brain barrier; SCL2A1 is responsible for the GLUT1 deficiency syndrome (infantile-onset epilepsy with heterogeneous type of seizures, complex movement disorders, ataxia, intellectual disability, macrocephaly, and hypoglycorrhachia) [66], and a large phenotypic spectrum characterized by normal glycorrhachia, movement disorders, often normal mental capacities, and seizures, in particular absence seizures [67]. Starting from these observation Suls et al. have found mutations in SCL2A1 gene in 12% of a cohort of 34 children with EOAE; the only clinical feature that can allow to differ children with EOAE from chidlren with CAE is the earlier age of onset. In addition, the authors preliminarily reported a marked reduction of epileptic activity at EEG in two of the mutated patients receiving ketogenic diet. In fact, it is of note that ketogenic diet may control seizures in cases of GLUT1 deficiency syndrome [65]. Lastly, a recent study performed by the same group of authors demonstrated that the epileptic phenotypic spectrum of GLUT1 deficiency is greater than previously recognized. In fact the authors have reported 12 patients with SCL2A1 mutations and epilepsy, including absence epilepsies with onset from early childhood to adult life, and various common forms of Idiopathic Generalized Epilepsy [68].

Recently, mutations in SLC2A1 gene leading to reduced function of GLUT1, the molecule transporting glucose across the blood-brain barrier, have been found in a significant proportion of children with absences starting before 4 years of age [65]. VPA monotherapy should represent the first-line treatment because it controls absences in the majority of cases [60,69]. Ethosuximide (ESM) had been previously used in some patients with a good seizure control [70]. Benzodiazepines are also effective to achieve seizure control, but they may induce tolerance and adverse effects [65,70].

### Panayiotopoulos Syndrome

Panayiotopoulos syndrome [PS] is characterised by seizures, often prolonged, with predominantly autonomic symptoms, and by an EEG that shows shifting and/or multiple foci, often with occipital predominance" [71]. PS occurs in children who are otherwise normal and, even after the most severe seizures and autonomic status epilepticus, the patient is normal and does not show any residual neurological or mental abnormalities. Accordingly, severe case of PS showed the apparent lack of consequences on the cognitive functions (Full Scale IQ = 106 at WISC-R) and normal sleep macrostructure profile in all night polysomnographic recording [72].

Previously considered as occipital epilepsy [73], PS has recently re-classified as "Autonomic Epilepsy" due to prominent autonomic manifestation which frequently represent the main (or sole) ictal epileptic semeiology [72,74-76].

Generally, age at onset is between 1 and 14 years (with 13% in the youngest age group of 3-6 years) and most individuals have their first seizure around the age of 5 years. This syndrome (affecting males and females of all races almost equally) is considered the most common of benign childhood partial seizures after Rolandic epilepsy.

Unilateral deviation of the eyes with or, rarely, without ictal vomiting is the commonest ictal manifestation after emesis, occurring in around two thirds of seizures (60%-80%). Seizures usually occur during nocturnal sleep, particularly the early part of sleep, or brief daytime naps. In nearly all seizures, consciousness is initially intact but becomes impaired as the seizure progresses, with the child becoming confused or unresponsive [77]. All functions of the autonomic nervous system can be affected, such as emesis, pallor, mydriasis, miosis, cardiorespiratory and thermoregulatory alterations, incontinence of urine and/or faeces, hypersalivation and modifications of intestinal motility; cardiorespiratory arrest and convulsive status epilepticus are exceptional [78].

The duration of the seizures is usually longer than 10 min [79]. Approximately half (44%) of them last from 30 min to many hours, developing an autonomic status epilepticus [75,79]. Interictal EEG shows occipital spikes (70%) although multifocal spikes with high amplitude sharp-slow wave complexes at various locations represent the most common EEG feature. Remarkably, the clinical manifestations are irrespective of EEG locations and despite similar clinical manifestations, there is a significant EEG variability from normal to abnormal EEG findings, sometimes in the same patient at different times. From a genetical point of view there are no available data, as well as about anticonvulsant therapy due to sporadic (and often not recognized) occurrence of seizures. Livingston JH et al described 2 families harboring a novel SCN1A mutation, one of whom had Panayiotopoulos syndrome and the other a phenotype consistent with generalized epilepsy with febrile seizures plus. The author argued that SCN1A mutations may cause susceptibility to an idiopathic focal epilepsy phenotype, the final phenotype depending on other (genetic or non genetic) factors [80]. Martín Del Valle F et al present two cases of Panayiotopoulos syndrome in two monozygotic twins, without SCN1A alteration, suggesting a genetic origin, with SCN1A associated with the outcome but not with the development of this syndrome [81].

As usually there is no need to start with anticonvulsant therapy, there are scarce available data about the efficacy of AEDs. In any case, recently, LEV showed good results in PS patients [82].

### Lennox-Gastaut Syndrome

As regards the etiology, LGS (a rare epileptic encephalopathy comprising generalized slow spikes-waves, mental deficiency and early onset of multiple and different seizures types) is usually divided into symptomatic or criptogenetic group, based on the presence or absence of neurological abnormalities or specific causes [83,84]. Symptomatic cases present different causes such as hypoxic-ischemic encephalopathy, vascular demage, perinatal meningoencephalitides, tuberous sclerosis, Down's Syndrome, trauma, brain tumor and malformations [83]; in the 10-25% of the patients a previous history of WS is reported [85].

Usually the onset of LGS is about 8 years of age and occurrence rates peak between 3 and 5 years. Clinically the type of seizures are different depending on the phases of the syndrome; tonic seizures are the most frequent and peculiar type of seizures (tonic axial seizures, particularly during sleep), but are not necessarily present at the onset. Tonic refers to "a sustained increase in muscle contraction lasting a few seconds or minutes". The patients can present flexor movement of head and trunk with apnoea and brief cry associated with abduction of the limbs, which usually involves the arms; in others patients the tonic seizures might involve not only trunkal muscles, and we can observe global tonic attacks [86]. The second seizure mainly associated with LGS is atypical absence, characterized by a brief loss of consciousness. Atonic falls or drop attacks are hazardous and occur in 56% of patients who have slow spike-waves, but are not diagnostic of LGS [87].

At EEG the characteristic and peculiar abnormalities are burst of diffuse slow spike-waves at 2-2,5 c/s during wakefulness, or burst or fast waves and slow polyspikes and generalized fast activity at about 10 c/s during sleep; this latter EEG finding is almost pathognomonic for LGS.

Developmental delay increases with time and the patients loose cognitive and intellectual skills. The prognosis of these patients is very poor and often their epilepsy remains untreatable [85].

Old AEDs for LGS treatment include benzodiazepine (CLB, CZP, diazepam (DZP), LZP, NZP), PB, Primidone (PRM), PHT, VPA, GVG [77]. Benzodiazepine are still prescribed with the specific risk in LGS of precipitating tonic status epileptic (TSE) [88]. Though they can control tonic-clonic seizures, PB and PRM should be avoided due to cognitive and sedative side effects [89]. PTH can control tonic-clonic seizures and reduce tonic seizures in LGS, but it can aggravate atypical absences and myoclonic seizures [84]. More recent therapeutic options in LGS are FBM, LTG, TPM and RUF [90,91]. FBM was the first to be approved for adjunctive therapy in LGS and showed a significant effect on "major" seizures. Severe adverse effects appeared a few months after approval and so, since 1994, FBM was administered exclusively for LGS refractory to other AEDs.

Antiepileptic and other drugs used "off-label" in LGS are acetazolamide (AZM), allopurinol, bromide, flunarizine, pyridoxine, ZNS [92].

LGS can occur in association with a variety of specific malformation of cortical development where Mendelian inheritance may be observed [93]. The occurrence in siblings is extremely rare and has been reported in a family with bilateral frontoparietal polymicrogyria due to GPR56 mutations [94]. Recently Lawrence et al reported a novel DXC mutation in a family with three siblings affected by LGS and anterior pachygyria [95].

### Benign childhood epilepsy with centrotemporal spikes (BCECTS)

Benign childhood epilepsy with centrotemporal spikes (BCECTS), or benign rolandic epilepsy (BRE), characteristically starts between 3 and 10 years of age, with a peak at 7 to 8, and resolves by puberty [96].

BCECTS, are usually brief, unilateral, tonic, clonic, or tonic-clonic, convulsions involving the face, lips, tongue, pharyngeal, and laryngeal muscles, with speech arrest, saliva pooling and drooling without loss of consciousness. The seizures usually occur during sleep and are sometimes followed by secondary generalization. The most common inter-ictal EEG features are centro-temporal diphasic or triphasic sharps or spikes occasionally spreading to the homologous contra-lateral region. BCECTS usually is associated with "idiopathic" conditions but it has also been reported with brain lesions such as cortical dysplasia [97]. Few ictal EEG in cases of rolandic epilepsy have been recorded; in these ceses the seizures begin with fast rhythms and spikes in the rolandic area that is contralateral to the clinical seizures, with a progressive increase in amplitude and admixture of slow waves or polyspikes waves.

Although BCECTS is considered a benign form of childhood epilepsy that occurs in children who show normal mental development, recent researches support the view that children with BCECTS show deficient performance in various neuropsychological areas, without a definition of a uniform profile [98,99].

On the other hand, the impact of epilepsy on cognitive function is complex, with many variables that can influence cognitive ability and interact, making it difficult to determine which factors contribute to the cognitive impairment [100]. In children with rolandic epilepsy, our group recently clearly indicate [98] an impairment in selectivity (impulsivity, focused attention, selective attention, aspects of divided attention) and in one measure of intensity (arousal) of attention whereas, the other measure of intensity of attention (vigilance) showed no impairment. Moreover, deficits of speech-related abilities seemed to be independent of the effects of antiepileptic treatment and are reversible after remission of epilepsy [99].

A strong genetic susceptibility has been suggested in BCECTS [101,102]. Very recently, a genome-wide study demonstrated linkage of the centrotemporal sharp wave EEG trait to 11p13 and several polymorphic markers in the ELP4 (Elongator Protein Complex 4) gene showed association with the BCECTS phenotype [103].

In contrast with most canonical forms of BCECTS, a rare syndrome combining the features of BCECTS and of speech and language disorders (rolandic epilepsy with speech dyspraxia, autosomal dominant: RESDAD) has shown monogenic inheritance [104]. Other families with monogenic inheritance of a syndrome with clinical features reminiscent, if not identical, to RESDAD and RESDX, have been described more recently [105]. Mutations in SRPX2 (Sushi-Repeat Protein, X-linked 2) cause rolandic epilepsy with speech impairment (RESDX syndrome) or with altered development of the speech cortex (bilateral perisylvian polymicrogyria) [106]. Comorbidity between BCECTS and altered development of speech motor control (also known as speech sound disorder) has also been further demonstrated in a recent case-control study [107**]**. Another BCECTS-associated genetic syndrome has been reported in a consanguineous family with rolandic epilepsy, paroxysmal exercise-induced dystonia, and writer's cramp. The syndrome is inherited as a recessive trait and linkage was found at 16p12-p11 [108], within the ''infantile convulsions and choreoathetosis (ICCA)'' critical region [109].

### Landau-Kleffner Syndrome

Landau Kleffner (LKS) is a rare syndrome of unknown etiology (more common in the children between 5 and 7 years af age) which consists mainly of loss of language skills in children previously normal [110,28]. Language regression may occur suddenly or in a prolonged period and aphasia can be primarily receptive or expressive; probably the language disorder can be defined as a verbal auditory agnosia, that consists of a loss of verbal comprehension, which may be confused as a acquired deafness [111]. This aphasia is followed by gradual deterioration also in verbal production and, finally, mutism and failure to respond to non-verbal sounds [111]. Associated with language disorder the children with LKS in the majority of cases present different types of seizures, which include episodes of eye blinking or ocular deviation, head drop and minor automatisms with secondary generalization. In other cases, sizures are focal or tonic-clonic generalized seizures, typical absences, partial complex and occasionally myoclonic seizures [112]. Early response to treatment and late outcome in LKS can be markedly different if the onset is in pre-linguistic phase (onset under the age of 3-4 years) and these children can be misdiagnosed for autistic due to impaired linguistic skills [113].

**Figure 2:**
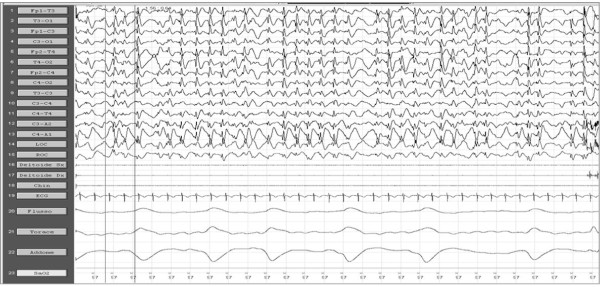
**Neuropolisomnography recording, before oral hydrocortisone administration**.

EEG shows bilateral temporal spikes or spikes waves, increasing during sleep. In these patients an awake EEG usually demonstrates normal background activity and focal epileptiform abnormalities especially on the temporal lobes; rarely awake EEG can be normal, making video-NPSG mandatory in these subjects. During the sleep EEG recording it is observed an activation and diffusion of the epileptic discharges, whereas the pattern of electrical status epilepticus during sleep (ESES or CSWS) may often be observed [111]. ESES picture is characterized by a continuous diffuse spike-waves during SS [114] (Figure 2) (see criteria definition for ESES in the next paragraph). ESES or CSWS presence is not mandatory to make diagnosis of LKS, although it is frequently associated [115].

Clinical seizures control in LKS is easy to obtain much more than ESES picture remission. Sleep EEG discharges in the majority of patients can persist months or years causing sometimes severe cognitive impairment, which can be transient, fluctuant or, rarely, irreversible with permanent cognitive deficits [100,111].

**Figure 3:**
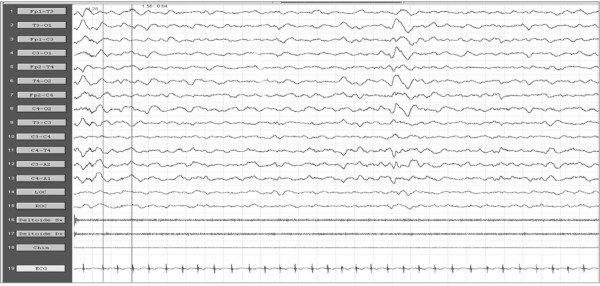
**After oral hydrocortisone administration, a complete remission of ESES activity was observed**.

Among available AEDs, CBZ may cause a worsening of seizures and it should be avoided. The treatment of choice in LSK appears to be VPA as mono-therapy or in combination with a benzodiazepine [28]. Some authors suggest that steroids and ACTH should be considered the treatment of choice especially in early onset of disease [116] with improvement in speech abilities [117] and EEG anomalies recovery (Figure 3).

CLB, NZP, VPA, ESM and flunitrazepam have been used with benefits. PB, CBZ, PHT have been reported to be ineffective or harmful [28]. Language functions and EEG abnormalities would be influenced significantly by intravenous immunoglobulin administration [118].

The prognosis of LKS is benign in most cases, although the improvement of language depends on age of onset (pre and post-linguistic onset) of the disorder and the severity of the epileptic seizures. Early onset makes the prognosis poorer with persistent language difficulties even in the adult life [112].

There is familial association that suggests a genetic factor in the development of both the epileptic discharges on EEG and cognitive dysfunction but there is no direct evidence of genetic predisposition featuring in LKS etiology [119].

LKS, the continuous spike-and-waves during sleep syndrome (ESES or CSWSS), and the BCECTS are different entities that are considered as part of a single continuous spectrum of disorders. Recent reports on the involvement of the SRPX2 and ELP4 genes with possible roles in cell motility, migration, and adhesion have provided first insights into the complex molecular bases of childhood focal epilepsies [120].

### ESES or CSWSS

ESES or CSWS is responsible for less than 1% of the age-dependent childhood EE, starting generally between the 5^th ^and 7^th ^year of life [121]. Even if these two terms are considered synonymous, ESES, firstly described by Tassinari [122], refers to the EEG pattern (continuous spike-wave complexes exclusively during non-REM sleep, with a spike-wave index accounting for at least 80-85% of SS), while CSWS indicates both EEG features and clinical neuropsychological characteristics of this EE [122,123]. From a clinical point of view various seizures type are possible in CSWS affected patient: generalized tonic-clonic seizures during sleep, atypical absence, myoclonic and atonic seizures. Developmental delay and deterioration resulting in a IQ reduction, loss of speech, behavior and motor involvement (with ataxia, dystonia, dyspraxia) is often associated [121,123]. In CSWSS may be present a natural history consisting in three phases: initial period with seizures and no developmental involvement (I); intermediate period with seizures, neuropsychological regression and ESES (II); final period with only neuropsychological deficits (III). However this evolution could be absent, and patients with CSWSS/ESES may present initially without seizures but "only" with developmental delay and/or behavior disturbance [123,124]. In these cases it could be useful to perform a video-NPSG investigation to deeper study the sleep EEG along the entire night [100].

A well-known partial epilepsy strongly related to ESES or CSWSS is the "Atypical Benign Partial Epilepsy of Infancy and Childhood" (ABPEI) also known as "Pseudo-Lennox Syndrome", firstly described by Aicard' e Chevrie [125]. It was often mistaken, in the past, for LGS because of the repeated atonic falls, absences and slow-wave activity at EEG. This form includes generalized seizures, atypical absences and atonic-astatic seizures. Axial tonic nocturnal seizures frequently observed in LGS are never observed in this type. ESES or CSWSS can be associated with ABPEI or even, in rare cases, with "Rolandic Epilepsy" [126].

Numerous AEDs have been used for the treatment of CSWSS, such as LTG, LEV, VPA, steroids, benzodiazepine; among the latter, high doses of DZP have been used with good results [121]. ESES or CSWSS pictures may be sometimes particularly refractory to AEDs and corticosteroid therapy (Figure 2 and 3) or AZM (12-15 mg/Kg/die), in these cases, have shown sometimes good results [100].

ACTH or hydrocortisone are effective, but side-effects are well-known. High doses of benzodiazepine (LZP, NZP) are effective when given in by rapid venous infusion or rectally. The rectal administration of DZP 1 mg/kg followed by an oral dose of 0.5 mg/kg/die for a period of 3 weeks gave positive results, with remissions lasting several months in 9/15 cases of ESES (60%) and in one typical case of LKF syndrome [125].

There is little evidence to suggest a genetic cause. Only 1 pair of monozygotic twins with CSWS has been reported. A family history of seizures, including febrile convulsions, is found in up to 15% of children with CSWSS [127]. A recent report of two families characterized by coexistent BCECTS and cryptogenic epilepsy with ESES in first-degree relatives suggest a possible genetic basis [128].

### Late-onset childhood occipital epilepsy (Gastaut type)

Gastaut type-idiopathic childhood occipital epilepsy (G-ICOE) or idiopathic childhood occipital epilepsy of late onset is a rare epileptic syndrome often with onset ranging from 3 to 15 years with a mean around 8 years of age. It is a pure form of idiopathic occipital epilepsy, included among the idiopathic focal epilepsies in childhood [1] with an uncertain long-term prognosis [129]. Seizure are purely occipital, frequent, brief and diurnal. They comprise simple partial seizures characterized by initial visual hallucinations (phosphenes and/or ictal blindness and illusions); ictal or postictal migraine headaches occur in half of the patients. Impairment of consciousness is rare unless associated with hemi-clonic or generalized convulsions [130]. Interictal EEG recordings reveal occipital spike and wave discharges that attenuate or disappear when the eyes are opened [131]. The prognosis is unclear, although remission occurs in 50-60% of patients within 2-4 years from onset. However, 40-50% of patients may continue to have visual seizures and infrequent secondary generalized tonic-clonic seizures, particularly if they have not been appropriately treated. Rarely, atypical evolutions to epilepsy with continuous spike-waves during slow wave sleep with cognitive deterioration have been reported [132].

Childhood occipital epilepsy-Gastaut type generally responds promptly to appropriate treatment with conventional AEDs, such as CBZ and VPA, with seizures usually remitting within 2-5 years. Verrotti et al. showed LEV as monohterapy to be effective and well tolerated [133].

The functional nature of this epilepsy syndrome was emphasized together with the presence of genetic predisposition in the affected patients. A possible autosomal dominant pattern for the EEG abnormalities with age dependent expression and variable penetrance of the seizure disorder has been proposed. Recent studies suggested an association between G-ICOE and idiopathic generalized epilepsies, suggesting that childhood absence epilepsy may have a closer genetic relationship to G-ICOE. There are reports about familial occurrence of G-ICOE [134].

### Jeavons Syndrome (Eyelid Myoclonia with Absences)

**Figure 4:**
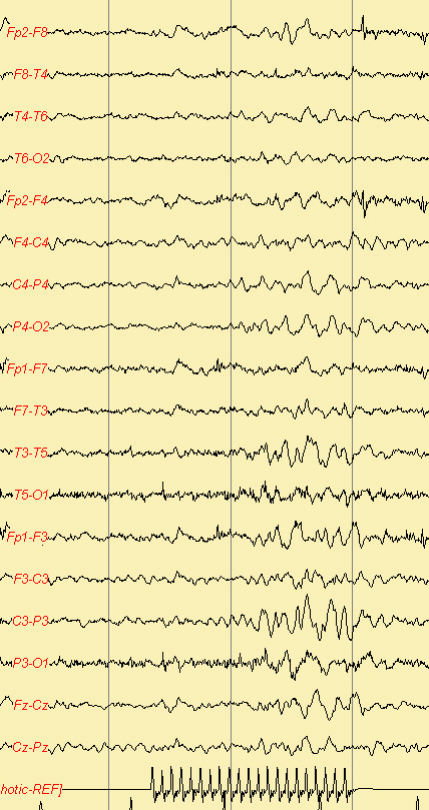
**Focal Photosensitive Paroxysmal Response**.

**Figure 5:**
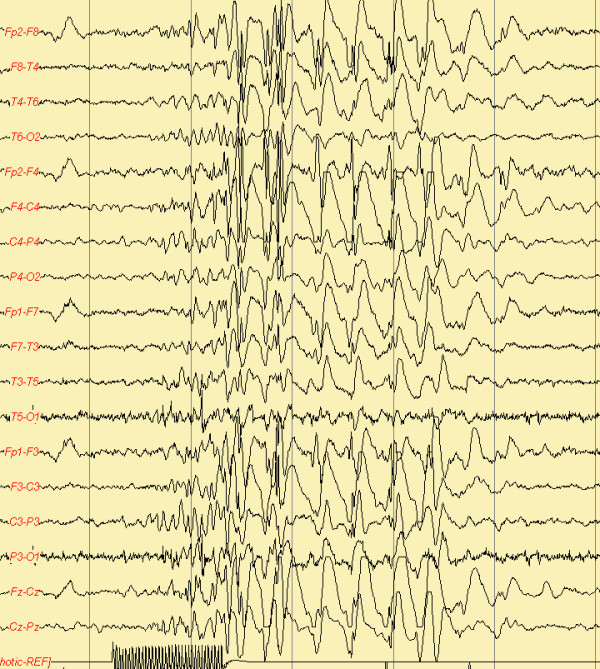
**Generalized Photosensitive Paroxysmal Response**.

Jeavons syndrome (JS) is one of the underreported epileptic syndromes characterized by eyelid myoclonia (EM), eye closure-induced seizures/electroencephalography (EEG) paroxysms, and photosensitivity (Figure 4 and 5). JS has been proposed as idiopathic generalized epilepsy (IGE) because of normal posterior dominant background activity and paroxysmal generalized ictal epileptiform discharges (EDs). The triad of manifestations are (1) eyelid myoclonia with and without absences; (2) eye closure-induced seizures and EEG paroxysms; and (3) photosensitivity [135].

Eyelid myoclonia, the hallmark of this syndrome, consists of marked jerking of the eyelids and often with jerky upward deviation of the eyeballs and the head (eyelid myoclonia without absences). This may be associated with or followed by mild impairment of consciousness (eyelid myoclonia with absences). The seizures are brief (3 to 6 sec) and occur mainly after eye closure and consistently many times per day. All patients are photosensitive. Generalized tonic-clonic seizures (GTCS), induced by lights or spontaneous, are probably inevitable in the long term and are particularly provoked by precipitating factors (sleep deprivation, alcohol) and inappropriate AEDs modifications. Typically, GTCS are sparse and avoidable. Myoclonic jerks of the limbs may occur but are infrequent and random. Eyelid myoclonic status epilepticus (1/5 of patients) consists of repetitive and discontinuous episodes of eyelid myoclonia with mild absence. Video-EEG is the most important procedure. It shows frequent and brief (2 to 3 sec) high-amplitude 3 to 6 Hz generalized spike and wave discharge of mainly polyspikes, which generally occur after eye closure. These are frequently associated with eyelid myoclonia. Photoparoxysmal responses occur in all untreated young patients. VPA, CNZ, ESM, and LEV are treatment options [135,136].

## Conclusions

There are some ECS with onset in paediatric age which can be associated with psychomotor development and cognitive skills deterioration; to prevent neuropsychiatric impairment, an early recognition can allow us to properly manage diagnostic flow-charts and appropriate therapeutic options,. Sleep interictal EEG discharges (even without associated diurnal or nocturnal clinical seizures) are able to cause transitory or permanent cognitive impairment, particularly in children with high rate of inter-ictal spiking. Cognitive functions, mainly under frontal lobe control, seem to be particularly vulnerable to epileptic EEG activity during the period of brain development and maturation. In this respect, from a physiopathological point of view, the most intriguing issue is represented by the relationship between ESES and the pattern of neuropsychological and/or motor derangement. Moreover, it become easy to understand how important is to search for new AEDs which will be able to achieve a better control of both, the clinical seizures and interictal paroxysmal EEG abnormalities, particularly during sleep.

In other cases, such as in Panayiotopoulos Syndrome, to avoid over-treatments, it is also crucial to know that even numerous long lasting "autonomic status epilepticus" episodes, seem to be associated with normal cognitive development. Yet, new advances on clinical natural course and slight cognitive impairment sometimes associated with BCECTS must warn about possible cognitive impairment in these epileptic children previously considered not at all at risks.

Finally, the knowledge of specific electro-clinical syndrome will help epileptologists to make the right diagnostic and therapeutic decisions for treating our children.

## Competing interests

All Authors report nor disclosures nor conflicts of interests in publishing this review.

## Authors' contributions

PP conceived of the study, and participated in its design, coordination and drafting/revising of the Review. AV, MCP, RC, FI, AF, FC and MPV participated in the design and drafting/revising of the review. All Authors read and approved the final manuscript
